# Computational Fluid Dynamic Technique for Assessment of How Changing Character of Blood Flow and Different Value of Hct Influence Blood Hemodynamic in Dissected Aorta

**DOI:** 10.3390/diagnostics11101866

**Published:** 2021-10-11

**Authors:** Andrzej Polanczyk, Aleksandra Piechota-Polanczyk, Ihor Huk, Christoph Neumayer, Julia Balcer, Michal Strzelecki

**Affiliations:** 1 The Faculty of Safety Engineering and Civil Protection, The Main School of Fire Service, 01-629 Warsaw, Poland; 2 Department of Medical Biotechnology, Faculty of Biochemistry, Biophysics and Biotechnology, Jagiellonian University, Gronostajowa 7 Street, 30-387 Krakow, Poland; aleksandra.piechota-polanczyk@uj.edu.pl; 3 Department of Surgery, Division of Vascular Surgery, Medical University of Vienna, Spitalgasse 23, 1090 Wien, Austria; ihor.huk@meduniwien.ac.at (I.H.); christoph.neumayer@meduniwien.ac.at (C.N.); 4 The Faculty of Electrical, Electronic, Computer and Control Engineering-Technologies, Lodz University of Technology, 93-924 Lodz, Poland; julia_balcer@wp.pl; 5 Institute of Electronics, Lodz University of Technology, 93-590 Lodz, Poland; michal.strzelecki@p.lodz.pl

**Keywords:** TEVAR, CFD, aortic dissection, blood flow reconstruction, medical image processing

## Abstract

Using computer tomography angiography (CTA) and computational structural analysis, we present a non-invasive method of mass flow rate/velocity and wall stress analysis in type B aortic dissection. Three-dimensional (3D) computer models of the aorta were calculated using pre-operative (baseline) and post-operative CT data from 12 male patients (aged from 51 to 64 years) who were treated for acute type B dissection. A computational fluid dynamics (CFD) technique was used to quantify the displacement forces acting on the aortic wall in the areas of endografts placement. The mass flow rate and wall stress were measured and quantified using the CFD technique. The CFD model indicated the places with a lower value of blood velocity and shear rate, which corelated with higher blood viscosity and a probability of thrombus appearance. Moreover, with the increase in Hct, blood viscosity also increased, while the intensity of blood flow provoked changing viscosity values in these areas. Furthermore, the velocity gradient near the tear surface caused high wall WSS; this could lead to a decreased resistance in the aorta’s wall with further implications to a patient.

## 1. Introduction

Acute aortic dissection is due to the separation of the layers of the aorta’s wall. A tear in the intimal layer results in the progression of the dissection (either proximal or retrograde) mainly due to the entry of blood between the intima and media [1]. A tear in the internal face of the aorta leads to dissection through the laminas and formation of a false lumen and acute drop in systemic blood pressure, potentially leading to hemopericardium and cardiac tamponade [2]. Two different classification systems are used by physicians to describe the location and extent of an aortic dissection [3]. The Stanford classification refers to dissections as Type A or Type B. Type A means that the dissection involves the ascending aorta, while in Type B it involves the descending aorta and/or abdominal aorta, without affecting the ascending aorta. The DeBakey classification system distinguishes between Type I, Type II, and Type III dissections. Type I involves the entire aorta, Type II involves the ascending aorta only, and Type III excludes the ascending aorta and aortic arch [4].

Several studies indicated that patient outcomes are improved when managed by an interprofessional team of healthcare professionals that includes a cardiologist, intensivist, pulmonologist, nephrologist, cardiac surgeon, interventional radiologist, and anesthesiologist [5]. Unless prompt surgical repair is performed, the patients usually die from complications related to the dissection, including rupture of the aorta, pericardial tamponade, aortic regurgitation, end-organ malperfusion, or acute heart failure [6]. Most dissections of the thoracic aorta present with acute symptoms, although the definition of acute aortic dissection is still debated [7]. Routine studies, e.g., an electrocardiography (ECG) or chest X-ray, may help differentiate other possible causes for chest pain; however, they may also be misleading. Furthermore, while the widening of the aortic silhouette increases the likelihood of acute aortic dissection, its absence does not reliably exclude the diagnosis. Confirmation of acute aortic dissection requires cardiovascular imaging to identify the presence of an intimal tear, establish the Stanford classification, and detect valvular or branch involvement [8]. A prompt diagnosis based on the early suspicion of aortic dissection is mandatory for successful treatment. However, the quickest and most accurate method to confirm the diagnosis is CT scanning of the aorta [9]. The sensitivity and specificity of CT are excellent, but in patients with poor renal function or allergy to iodinated dye, CT angiography is questionable, and other imaging modalities such as magnetic resonance imaging (MRI) or ultrasonography should be considered instead [10].

The current in-hospital mortality rates remain significant, with reported mortality rates of approximately 10% [11]. For this reason, computational cardiovascular mechanics has allowed scientists to create complex 3D models for the simulation of cardiovascular problems [12]. The application of the computational fluid dynamic (CFD) technique in the topic of blood hemodynamic in vessels is widely described in the literature [13,14]. The real three-dimensional geometry of vessels is usually reconstructed with the use of medical data acquired from CT or magnetic resonance imaging [15]. CFD models of blood flow in simplified aorta mathematical models have been studied with homogeneously and symmetrically distributed velocity [16]. However, such geometries may not accurately represent the complicated patient-specific aorta, as the wall shear stress (WSS) is severely impacted by the topological structure of the aortic wall when comparing idealized and realistic aorta models [17]. Therefore, CFD simulations based on patient-specific models may provide insight into the biomechanical behavior of blood flow in the type B dissection, allow quantitative analysis of hemodynamic patterns, and predict clinical progression of aortic dissection; however, its clinical value remains to be verified. The application of computational methods allows different mechanical and fluid parameters analyses, e.g., wall shear stress is the force parallel to the aortic wall that flowing blood exerts. Shear stresses have been shown to affect endothelial cell functions such as proliferation, migration, and remodeling, as well as platelet activation [18]. Additionally, in our previous research we focused on brightness analysis for the patients with aortic wall dissection. It was noticed that each time a connection of the true and false duct appeared, the true duct had lower brightness compared to the common duct and false duct. Also, false duct was characterized by higher brightness if compared to common duct [19]. Therefore, the aim of the study was to prepare a CFD model of chosen blood flow parameters within the aorta and aortic branches in patients with type B aortic dissection before and after a thoracic endovascular aortic repair procedure. This may enable physicians to predict chronic aneurysmal degeneration in patients who are medically managed for acute type B dissections.

This paper is organized as follows: In Section 2, medical data and a mathematical model with boundary conditions and its verification is described. Section 3 presents the results directed in the computer simulation. In Section 4, a discussion is proposed, while Section 5 concludes the paper.

## 2. Materials and Methods

In our study, image data from 12 patients (men, aged from 51 to 64 years) after computed tomography angiography (CTA) (GE Light-Speed 64 VCT; GE Healthcare, Fairfield, CT, USA) as well as blood hemodynamic from USG-Doppler (GE Vivid 7, GE Healthcare, Fairfield, CT, USA) was collected (Table 1). All 12 patients underwent treatment with vascular and endovascular procedures at the Medical University of Vienna between 2012 and 2014. The study was approved by the local Institutional Review Board (2069/2012) of the Medical University of Vienna. The aortic reconstruction comprised implantation of a stent-graft in the distal aortic arch and the descending thoracic aorta (LSA covered), and depending on the analyzed patient, the implantation of self-expanding stents into the left renal artery or the right common iliac artery.

**Figure 1:**
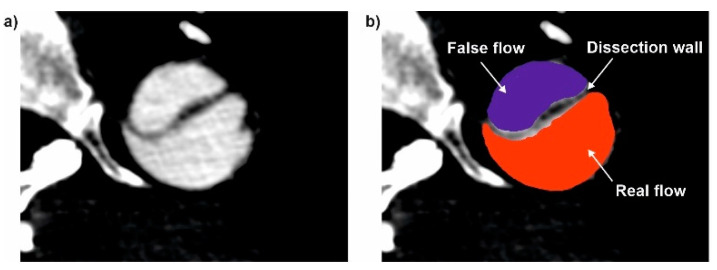
An example of (**a**) DICOM with (**b**) indication of False and Real flow and dissected wall.

First, the 3D reconstruction of a virtual model of an aorta with a dissected wall and after surgical intervention was made. Anonymized pre-operative (baseline) and post-operative AngioCT data (512 × 512 × 270 voxels, in-plane resolution of 0.78 × 0.78 mm, slice thickness 1 mm) from the patients with acute complicated type B dissection formed the base for this study. Digital Imaging and Communications in Medicine (DICOM) data before thoracic endovascular aortic repair and after thoracic endovascular aortic repair were analyzed. Each time, a 3D model of the investigated part of the human vascular system (from aorta’s arch to femoral aorta’s) was prepared with the usage of AngioCT data (Figure 1).

**Figure 2:**
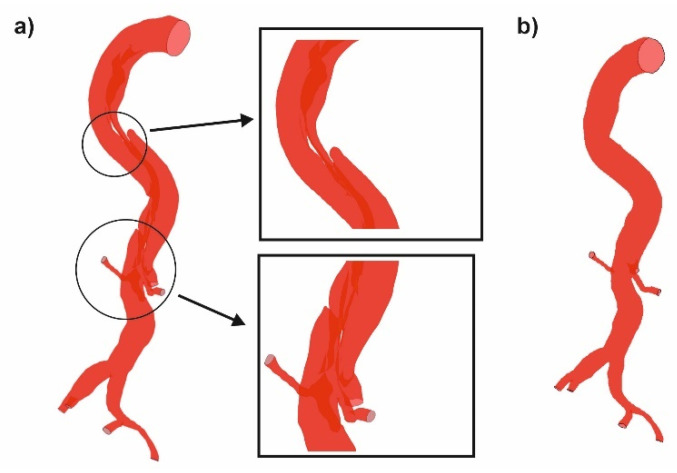
Investigated part of human vascular system (**a**) with the aorta’s wall dissection, (**b**) without aorta’s wall dissection.

To investigate how the dissection of an aorta’s wall influences the blood hemodynamic, Angio CT data from 12 patients was applied and considered in two cases, before and after surgical intervention, with (Figure 2a) and without (Figure 2b) the aorta’s wall dissection, respectively. Each time DICOM data was applied to extract a model of the aorta to generate the surface object, stored in the stereolithography (STL) format. To achieve the highest contrast between analyzed objects (aorta and surrounding tissues), AngioCT data was manually adjusted for brightness. Moreover, to extract the aorta from the background, the region growing technique was applied. ImageJ software and its tool for morphological hole-filling was used for gap elimination. The implemented segmentation region growing technique provided accurate results, since the aorta gray levels differed significantly from the image background. When compared to manual segmentation performed by the radiologist, the estimated aortas did not differ more than by 5%. For each reconstructed 3D model of the aorta after the segmentation process, a rendering was performed. Moreover, a quantitative analysis of AngioCT data following [21] was used. Brightness to noise (BI), a quotient of analyzed object brightness value and a parameter that defines noise intensity, was calculated. Secondly, contrast to noise ratio (CNR) as a quotient of subtraction of the analyzed object and background brightness to noise intensity was computed [22]. CNR was calculated by placing the region of interest (ROI) in the center of the area represented by the analyzed aorta (reaching 94 ± 3.2 mm^2^), which corresponds to the image fragment with maximum brightness. This operation was performed for all slices of each patient. The mean of these values was used for further calculations. Image noise was parametrized as a ratio of ROI and standard deviation measured in pixels and calculated for 100 mm^2^, drawn in two different regions located in the image background (regions without the signal) left and right sides. The brightness to noise was equal to 27.85 ± 1.4, while average contrast to noise ratios was equal to 3.65 ± 0.7.

**Figure 3:**
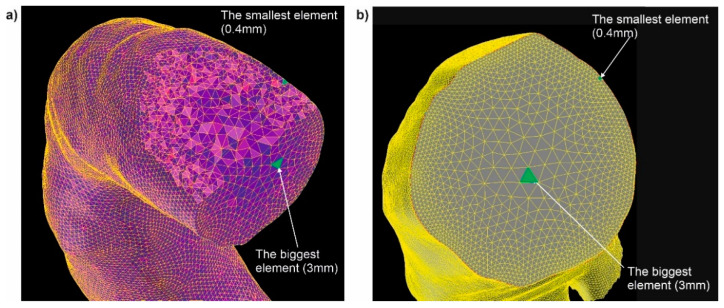
The size of elements inside analyzed part of human vascular system: (**a**) aorta with wall dissection, (**b**) aorta without wall dissection.

At the beginning, discretization was performed with the use of ANSYS ICEM CFD (ANSYS, Canonsburg, PA, USA) [23]. Volume meshing with tetrahedral elements type T-Grid was applied (Figure 3a,b) [24]. The size of elements ranges from 0.4 to 3 mm for each analyzed aorta with wall dissection (Figure 3a), and from 1 to 3 mm for each aorta without wall dissection (Figure 3b). The numerical grids were composed of approximately 5,000,000 tetrahedral elements with a boundary layer composed of prism elements next to the wall. The density of layers of mesh gradually decreases from the adjacency of the wall to the inner fluid domain, with the first layer mesh thickness of 0.1 mm and the expanding ratio of 1.15. Analogous calculations were performed for the dissection area where prism elements were applied for the boundary layers. To neglect the influence of the size and/or number of numerical grid elements on the results of computer simulation, a mesh independent test was performed. Initially, mesh independent testing was performed for different sized grid elements. The tested range of elements for the whole analyzed domain was equal to 0.4 mm–5 mm (with an expanding ratio equal to 1.15), decreasing the size of elements to 0.4 mm around the wall. It was observed that for an element size of 5 mm and 4 mm, the model failed to converge on a solution. However, for the meshes composed of 3 mm and smaller elements, the CFD model simulations converged on a solution. Thus, to minimize the size of the numerical grid as well as minimize the time taken for calculations, the final mesh, depending on the analyzed case, consisted of around 5,000,000 tetrahedral elements, including the boundary layer (Figure 3).

In the next step, a computational fluid dynamic (CFD) technique was applied for the reconstruction of real blood flow and WSS distribution for one cardiac cycle as previously described [25]. ANSYS FLUENT 19.2 software (ANSYS, Canonsburg, PA USA) was applied for blood hemodynamic reconstruction in the analyzed domains as previously described [26]. A numerical simulation of pulsatile blood flow through the aorta model was conducted by directly solving the Navier-Stokes equations (Equations (1) and (2)).

(1) $$\rho \left(\frac{\partial u}{\partial t}+u\cdot \nabla u\right)=-\nabla p+\mu \nabla^{2}u$$

(2) ∇·u=0

where the symbol *u* represents the blood velocity vector, *p* represents blood pressure, *ρ* represents blood density, and *µ* represents blood viscosity (Equation (3)).

**Figure 4:**
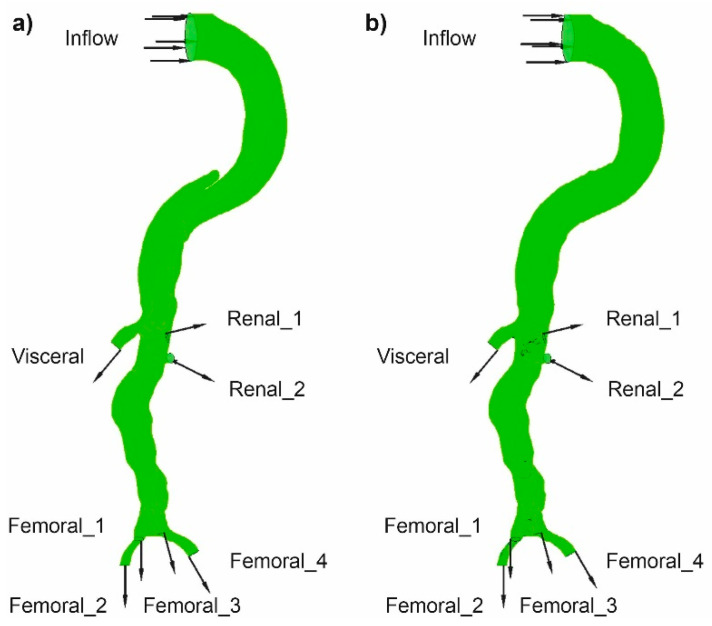
Mathematical domains of the analyzed part of the human vascular system (**a**) with aorta’s wall dissection, (**b**) without aorta’s wall dissection. Inlet (Inflow) and outlet (Visceral, Renal, Femoral) areas were marked.

Blood flow was incompressible and laminar. The following boundary conditions were applied for the description of the analyzed mathematical domain: inlet was described with the use of velocity-inlet (*v*(*x,y,z*)), outlets were described with the pressure conditions, and the wall was treated as a rigid structure (Figure 4). Moreover, a blood velocity profile from USG traces (GE Vivid 7, GE Healthcare, USA) [27] before and after surgical intervention was set at the inlet, while at the outlets (Visceral, Renal_1, Renal_2, Femoral_1, Femoral_2, Femoral_3, Femoral_4) routine blood pressure value for the certain vessel type was set. Each time, three profiles of blood velocity were applied to examine how the investigated part of the human vascular system behaves for different characters of blood flow (two medium—measured in a patient before and after surgical intervention and high intensity—virtually reconstructed higher intensity of blood flow). The rheological properties of blood were described with the use of a modified Quemada’s model (Equations (3) and (4)), as previously described [28,29]. To investigate how a changing value of viscosity influences blood hemodynamic, two *Hct* values (*Hct* = 40%—measured in patient and *Hct* = 50%—simulating higher resistance for blood flow) were analyzed. Blood density was assumed as a constant value of 1040 kg/m^3^ [30].

(3) $$\mu =\mu_{p}{\left(1-\frac{K\cdot Htc}{2}\right)}^{-2}$$

(4) $$K=\frac{k_{0}+k_{\infty }{\left(\gamma /\gamma_{c}\right)}^{1/2}}{1+{\left(\gamma /\gamma_{c}\right)}^{1/2}}$$

where the symbol *μ_p_* represents plasma viscosity, *Htc* ishematocrit, *K* represents the inner viscosity of erythrocytes (Equation (4)), *k*_0_, *k_∞_* represent parameters characterizing blood behavior, *γ* is the shear thinning rate, and *γ_c_* is the critical value of the shear rate.

The cardiac cycle for this study was equal to 1 s. First, it was extracted from ten consecutive cycles, each of which measured 1 s. Then, the three first cycles were excluded according to Tyfa et al. [31].

**Figure 5:**
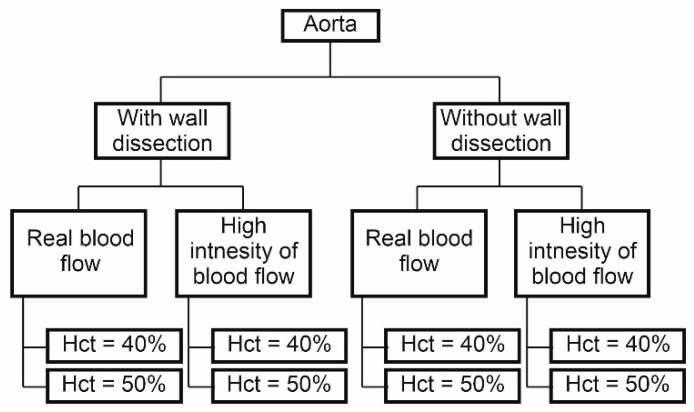
Initial hemodynamic conditions for analyzed cases.

The influence of three parameters on blood hemodynamics in the dissected aorta was investigated: (1) spatial configuration of the wall, (2) blood viscosity, and (3) blood flow intensity (Figure 5). The spatial configuration of the aorta wall, especially during flow through the narrow parts of aorta’s wall dissection, before and after surgical intervention was analyzed. The influence of blood viscosity was included with a changing value of *Hct*. Finally, two blood velocity profiles in function of time were applied. Thus, calculations were performed for eight cases for each patient; four were performed for the aorta with wall dissection, and another four for the aorta without wall dissection (Figure 5).

Statistical analysis was performed with the use of Statistica 12 software. Values were presented as mean ± SD. Comparison between pre- and post-operative patients were made with Student’s test after verifying normality and variance. Moreover, USG data recorded before and after surgical intervention from the outlets of the analyzed circulatory system were applied for the process of mathematical model verification and confronted with CFD results. Furthermore, the Bland-Altman method was applied to analyze the agreement between medical data and CFD results.

## 3. Results

Blood distribution for the investigated part of the human vascular system (from aorta’s arch to femoral arteries) was described with the use of the following parameters: blood velocity, pressure, viscosity, and wall shear stress (WSS) during pulsating flow. Simulations were performed for each patient for eight cases to investigate how a changing character of blood flow and changing geometry of blood flow channel correlate with different values of *Hct*, and how they influence blood hemodynamic in dissected and non-dissected aorta.

### 3.1. Blood Velocity

**Figure 6:**
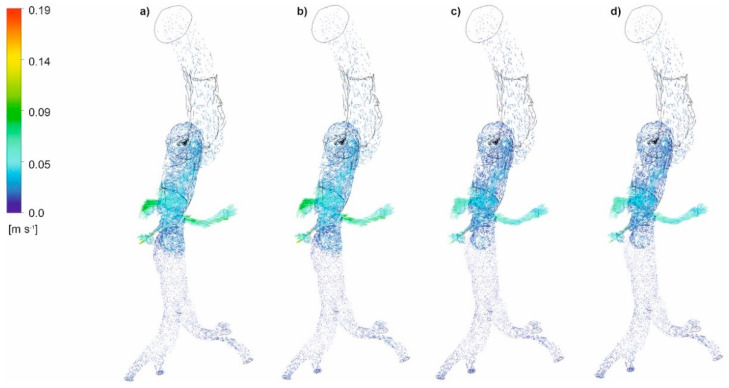
Velocity vectors for an aorta with dissection (**a**) *Hct* = 0.4 and high intensity of blood flow, (**b**) *Hct* = 0.5 and high intensity of blood flow, (**c**) *Hct* = 0.4 and real intensity of blood flow, (**d**) *Hct* = 0.5 and real intensity of blood flow; timestep 3.18 s; values presented as (m/s).

**Figure 7:**
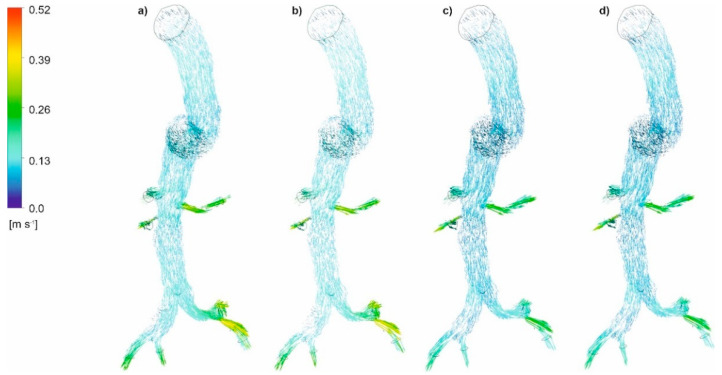
Velocity vectors for an aorta without dissection (**a**) *Hct* = 0.4 and high intensity of blood flow, (**b**) *Hct* = 0.5 and high intensity of blood flow, (**c**) *Hct* = 0.4 and real intensity of blood flow, (**d**) *Hct* = 0.5 and real intensity of blood flow; timestep 3.18 s; values presented as (m/s).

The distribution of blood velocity vectors for an aorta with and without wall dissection for a changing *Hct* value and blood flow intensity was first analyzed. Blood velocity vector distribution for the representative patients for both cases is presented in Figure 6a–d (with aortic dissection) and Figure 7a–d (without aortic dissection). For both cases, with and without wall dissection, the highest blood velocity was observed for *Hct* = 40% and high intensity of blood flow. Moreover, with an increase in blood flow and the *Hct* value, a change in the location of maximum velocity vectors was observed. Furthermore, low blood velocity value was noticed within the proximal false lumen, with slow flow recirculation at the upper right adjacent to the first entry tear. Flow accelerations were observed in bending areas.

A comparison of the thoracic trunk for both cases indicated that an aorta with wall dissection had lower blood velocity comparing to the aorta without wall dissection (24% and 23% decrease of blood velocity for real and higher intensity of blood flow for *Hct* = 40%, respectively). An increase in the *Hct* value to 50% caused a 3% increase in blood velocity. A comparison of the brachiocephalic trunk for both cases indicated that wall dissection resulted in lower blood velocity compared to the cases without wall dissection (14% and 15% decrease in blood velocity for real and higher intensity of blood flow for *Hct* = 40%, respectively). Additionally, an increase in the *Hct* value to 50% resulted in a 14% decrease in blood velocity. A comparison of the carotid arteries indicated that wall dissection led to lower blood velocity compared to the cases without wall dissection (6% decrease in blood velocity for real and higher intensity of blood flow for *Hct* = 40%). For an *Hct* = 50%, a 6% decrease in blood velocity was observed. Further comparison of subclavian arteries for both cases indicated that wall dissection resulted in lower blood velocity compared to the cases without wall dissection (6% and 7% decrease in blood velocity for real and higher intensity of blood flow for *Hct* = 40%, respectively). For *Hct* = 50%, a 7% and 8% decrease in blood velocity for real and higher intensity was observed. Moreover, a 14% decrease in blood velocity for real and higher intensity of blood flow was observed in the femoral arteries (for *Hct* = 40%). An increase in the *Hct* values reduced the blood flow velocity to 12% (higher intensity of blood flow) and 11% (real blood velocity), respectively.

**Figure 8:**
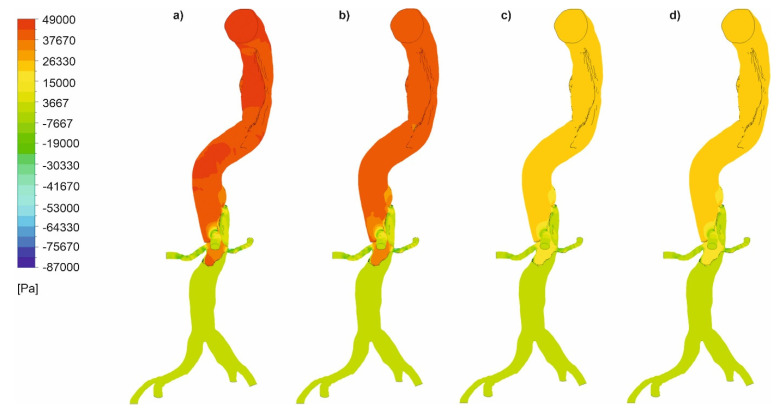
Static pressure along an aorta with dissection of a wall, (**a**) *Hct* = 0.4 and high intensity of blood flow, (**b**) *Hct* = 0.5 and high intensity of blood flow, (**c**) *Hct* = 0.4 and real intensity of blood flow, (**d**) *Hct* = 0.5 and real intensity of blood flow; timestep of 3.18 s; values presented as (Pa).

**Figure 9:**
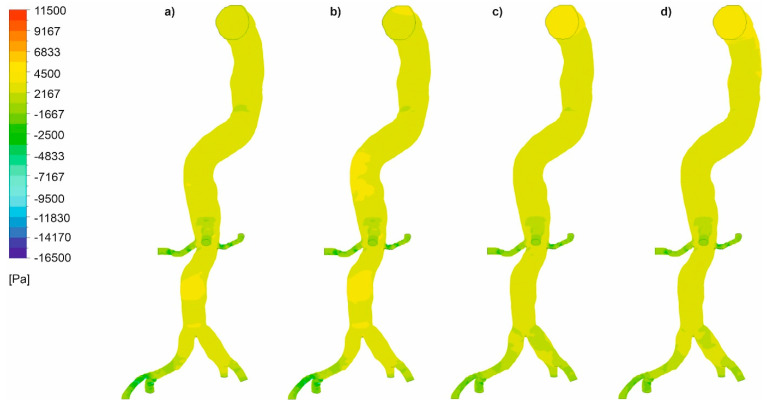
Static pressure along an aorta without dissection of a wall, (**a**) *Hct* = 0.4 and high intensity of blood flow, (**b**) *Hct* = 0.5 and high intensity of blood flow, (**c**) *Hct* = 0.4 and real intensity of blood flow, (**d**) *Hct* = 0.5 and real intensity of blood flow; timestep of 3.18 s; values presented as (Pa).

In the next step, we analyzed pressure distribution for the whole mathematical domain. It was observed that static pressure contours for the cases with wall dissection (Figure 8) and without wall dissection (Figure 9) reflected blood velocity distribution as described above.

**Figure 10:**
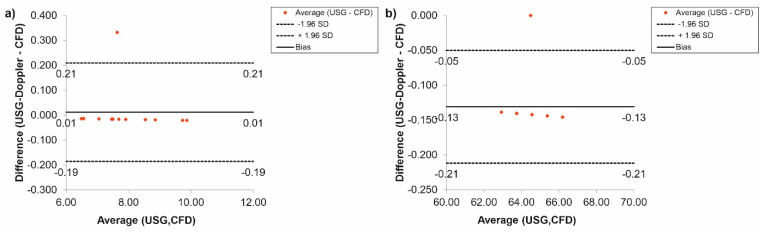
Comparison of CFD and USG-Doppler measurement for the thoracic trunk with the use of Bland-Altman analysis for: (**a**) patients with aortic dissection, (**b**) patients without aortic dissection.

Furthermore, the CFD results regarding blood flow were verified with the use of USG-Doppler measurements. There were no significant changes between results gathered from CFD simulations and USG-Doppler measurements (Figure 10). The Bland-Altman analysis showed that in the case of the thoracic trunk, for *Hct* = 40% before TEVAR, the difference between CFD and USG-Doppler was equal to 0.01 mL/s for range 0.40 mL/s (Figure 10a), while after TEVAR, the difference was equal to 0.13 mL/s for the range 0.26 mL/s (Figure 10b).

**Figure 11:**
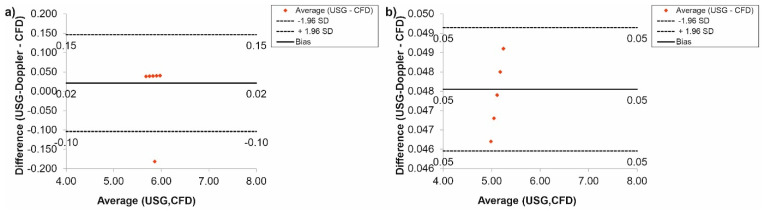
Comparison of CFD and USG-Doppler measurement for the brachiocephalic trunk with the use of Bland-Altman analysis for: (**a**) patients with aortic dissection, (**b**) patients without aortic dissection.

Additionally, the Bland-Altman analysis of the brachiocephalic trunk, for *Hct* = 40% before TEVAR showed that the difference between CFD and USG-Doppler was equal to 0.02 mL/s for range 0.25 mL/s (Figure 11a), while after TEVAR, the difference was equal to 0.05 mL/s for the range 0.10 mL/s. (Figure 11b).

**Figure 12:**
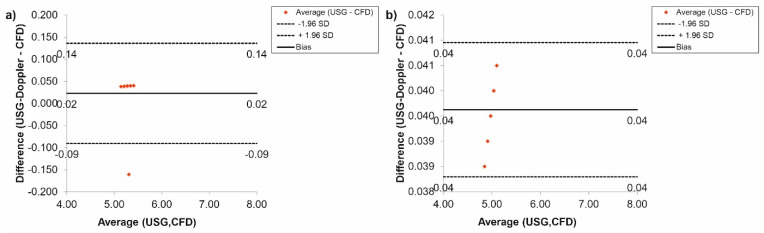
Comparison of CFD and USG-Doppler measurement for the carotid arteries with the use of Bland-Altman analysis for: (**a**) patients with aortic dissection, (**b**) patients without aortic dissection.

In the case of the carotid arteries, the Bland-Altman analysis showed that for *Hct* = 40% before TEVAR, the difference between CFD and USG-Doppler was equal to 0.02 mL/s for range 0.23 mL/s (Figure 12a), while after TEVAR, the difference was equal to 0.04 mL/s for the range 0.08 mL/s. (Figure 12b).

**Figure 13:**
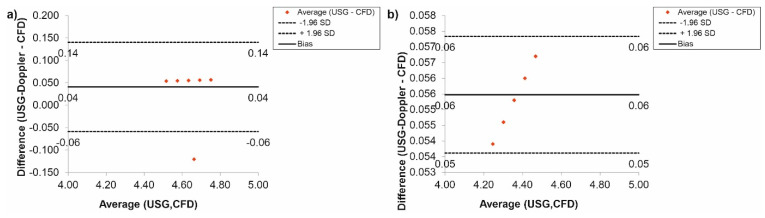
Comparison of CFD and USG-Doppler measurement for the subclavian arteries with the use of Bland-Altman analysis for: (**a**) patients with aortic dissection, (**b**) patients without aortic dissection.

The Bland-Altman analysis of the results gathered for the subclavian arteries, for *Hct* = 40% before TEVAR, showed that the difference between CFD and USG-Doppler was equal to 0.04 mL/s for range 0.20 mL/s (Figure 13a), while after TEVAR, the difference was equal to 0.06 mL/s for the range 0.11 mL/s. (Figure 13b).

**Figure 14:**
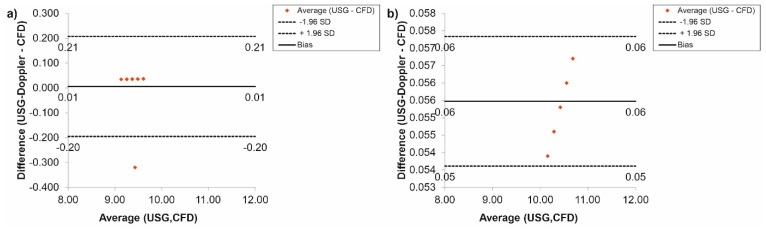
Comparison of CFD and USG-Doppler measurement for the renal arteries with the use of Bland-Altman analysis for: (**a**) patients with aortic dissection, (**b**) patients without aortic dissection.

Next, the Bland-Altman analysis used for results gathered for the renal arteries, for *Hct* = 40% before TEVAR, indicated that the difference between CFD and USG-Doppler was equal to 0.01 mL/s for range 0.40 mL/s (Figure 14a), while after TEVAR, the difference was equal to 0.06 mL/s for the range 0.11 mL/s. (Figure 14b).

**Figure 15:**
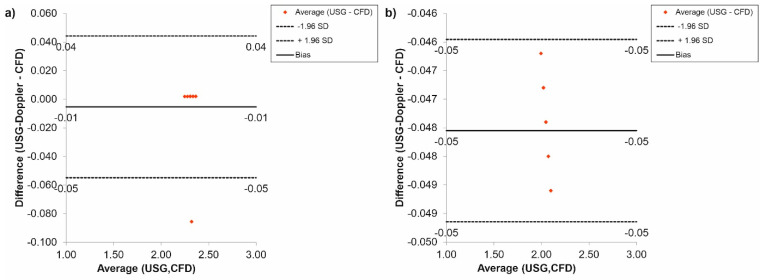
Comparison of CFD and USG-Doppler measurement for the femoral arteries with the use of Bland-Altman analysis for: (**a**) patients with aortic dissection, (**b**) patients without aortic dissection.

Finally, according to a Bland-Altman analysis of the results for the femoral arteries, for *Hct* = 40% before TEVAR, the difference between CFD and USG-Doppler was equal to 0.01 mL/s for range 0.10 mL/s (Figure 15a), while after TEVAR, the difference was equal to 0.05 mL/s for the range 0.10 mL/s. (Figure 15b).

### 3.2. Wall Shear Stress

**Figure 16:**
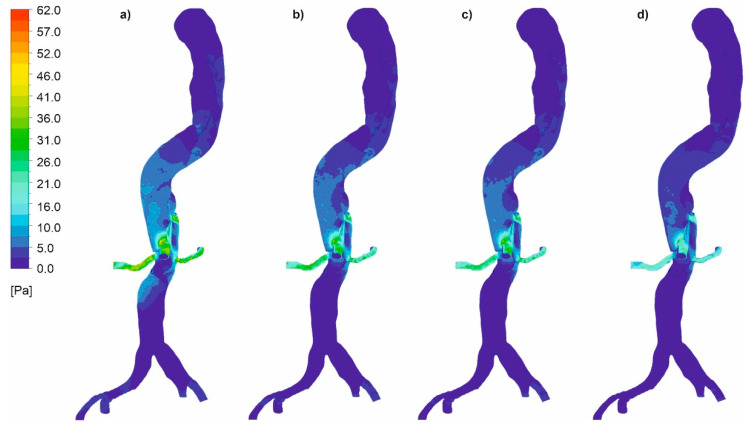
Wall shear stress along an aorta with dissection of wall, (**a**) *Hct* = 0.4 and high intensity of blood flow, (**b**) *Hct* = 0.5 and high intensity of blood flow, (**c**) *Hct* = 0.4 and real intensity of blood flow, (**d**) *Hct* = 0.5 and real intensity of blood flow; timestep was 3.18 s; values presented as (Pa).

**Figure 17:**
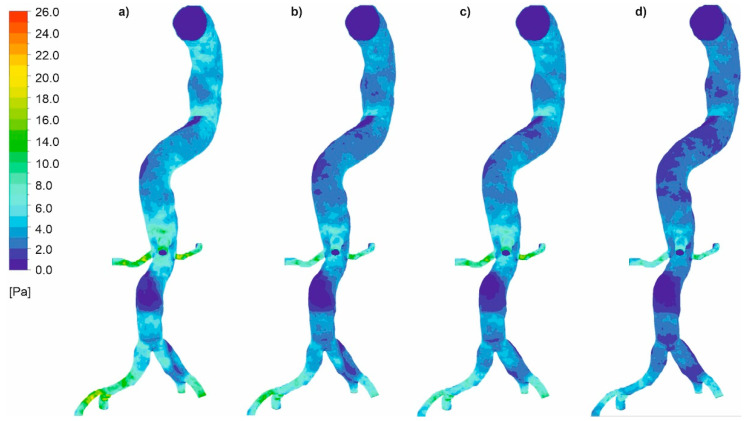
Wall shear stress along an aorta without dissection of wall, (**a**) *Hct* = 0.4 and high intensity of blood flow, (**b**) *Hct* = 0.5 and high intensity of blood flow, (**c**) *Hct* = 0.4 and real intensity of blood flow, (**d**) *Hct* = 0.5 and real intensity of blood flow; timestep was 3.18 s; values presented as (Pa).

**Figure 18:**
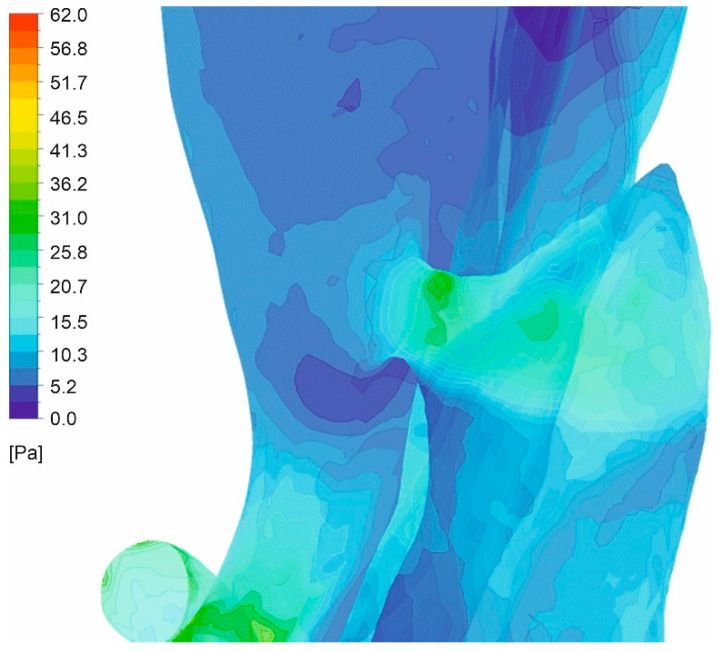
WSS distribution near a gap connection between the false and real flow (high intensity of blood flow *Hct* = 0.4).

Finally, the distribution of wall shear stress contours for an aorta with and without wall dissection for a changing *Hct* value and blood flow intensity was analyzed. Wall shear stress distribution for the representative patient for both cases was presented in Figure 16a–d (with aortic dissection) and Figure 17a–d (without aortic dissection). Moreover, the distribution near a gap connection between false and true lumen was presented in Figure 18. The results indicated that for both cases, with and without dissection, the highest wall shear stress was observed for *Hct* = 40% (average value was equal to 3.79 ± 0.55 Pa and 1.30 ± 0.19 Pa for the case with aortic dissection and without aortic dissection, respectively).

A comparison of the thoracic trunk for both cases indicated that cases with wall dissection were characterized by higher average wall shear stress (3.15 ± 0.26 Pa and 2.64 ± 0.24 Pa for *Hct* = 40% and *Htc* = 50%, respectively) compared to the cases without wall dissection (1.34 ± 0.13 Pa and 1.12 ± 0.11 Pa *Hct* = 40% and *Htc* = 50%, respectively). Moreover, further analysis of subsequent arteries, i.e., the renal arteries, indicated that a case with wall dissection presented higher average wall shear stress (4.36 ± 0.24 Pa and 3.65 ± 0.22 Pa for *Hct* = 40% and *Htc* = 50%, respectively) compared to the cases without wall dissection (1.38 ± 0.23 Pa and 1.16 ± 0.20 Pa *Hct* = 40% and *Htc* = 50%, respectively). Also, comparison of the femoral arteries for both cases indicated that cases with wall dissection had higher average wall shear stress (3.86 ± 0.16 Pa and 3.23 ± 0.11 Pa for *Hct* = 40% and *Htc* = 50%, respectively) compared to the cases without wall dissection (1.17 ± 0.12 Pa and 0.98 ± 0.10 Pa *Hct* = 40% and *Htc* = 50%, respectively). Moreover, it was observed that the velocity gradient near the tear surface caused high WSS. Also, the existence of multiple distal tears allowed blood flow to alternatively enter or exit the false lumen.

**Figure 19:**
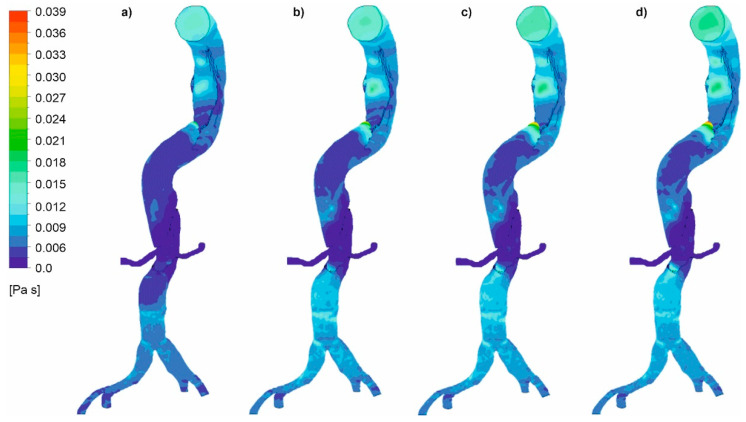
Viscosity distribution for a case with wall dissection for: (**a**) high intensity of blood flow and *Hct* = 0.4, (**b**) high intensity of blood flow and *Hct* = 0.5, (**c**) real intensity of blood flow and *Hct* = 0.4, (**d**) real intensity of blood flow and *Hct* = 0.5; timestep 3.18 s; values presented as (Pas).

**Figure 20:**
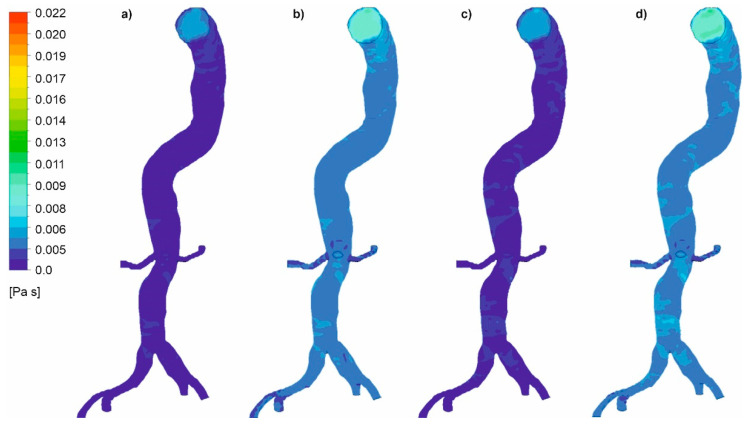
Viscosity distribution for a case without wall dissection for: (**a**) high intensity of blood flow and *Hct* = 0.4, (**b**) high intensity of blood flow and *Hct* = 0.5, (**c**) medium intensity of blood flow and *Hct* = 0.4, (**d**) medium intensity of blood flow and *Hct* = 0.5; timestep 3.18 s; values presented as (Pas).

**Figure 21:**
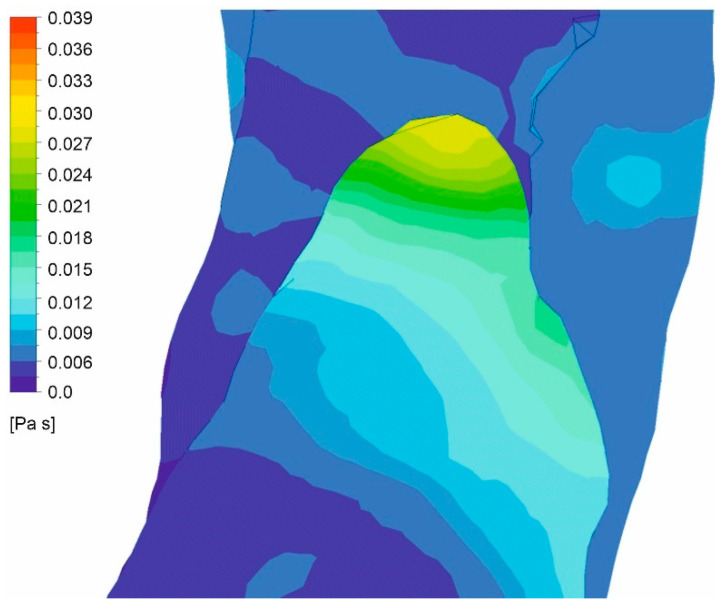
Viscosity distribution near area of low blood velocity (high intensity of blood flow *Hct* = 0.4).

Moreover, viscosity distribution was analyzed for both cases. It was observed that with an increasing value of *Hct*, which reflects an increase in blood viscosity (Figure 19 and Figure 20), average and maximum values of WSS decreased. Finally, for wall dissection, there were places in the upper part of the aorta where velocity vectors had lower values compared to the case without dissection, which provoked an increase in blood viscosity (Figure 21) and decrease in WSS.

Furthermore, near the places where true and false lumen connected, the value of WSS increased. A higher value of WSS may have involved an increased size in the false and real blood flow connection gap, which could lead to decreased resistance in the aorta’s wall.

## 4. Discussion

Acute complications may require urgent intervention. However, most patients are discharged without intervention. Despite optimal medical management, a subset of these patients will develop aneurysmal degeneration [11]. Our previous results showed that each time a connection of the true and false duct appeared, the true duct had lower brightness compared to the common duct and false duct, and the false duct was characterized by higher brightness [19]. Current results indicated that the parameters derived from CFD simulations on patient-specific aortic dissection geometries strongly correlate to medical data derived from such patients. The CFD was applied to assess how the morphologic variation of acute type B dissections affects the flow and wall shear stress patterns.

Karmonik et al. examined the evolution of a single patient’s type B dissection over time, showing decreasing total pressure and time-averaged wall shear stress over time as the false lumen gradually thrombosed [32].

Additionally, it was observed that static pressure was the highest at the graft location due to narrowing at the aortic arch. It was in line with Tsai et al., who noticed that pressure in the true lumen is higher than that in the false lumen proximally, while the opposite happens in the distal region [33]. Similarly, Naim et al. described that higher pressure in the true lumen may prevent compression of the true lumen, whereas higher pressure in the false lumen might promote its dilation [34]. Moreover, WSS for true lumen was higher than that in the false lumen. This is in line with a study by Rinaudo and Pasta, who noticed that all tear regions experience typical high time averaged wall shear stress, which may indicate the potential for tear propagation [35]. Furthermore, to achieve realistic WSS values, we decided to describe blood rheological properties with a non-Newtonian model. This approach was in line with Xiang et al., who showed that non-Newtonian fluid reflects the real properties of blood, and does not artificially increase WSS as the Newtonian approach does [36].

Moreover, our data show that higher blood flow was observed for the false lumen compared to the true lumen. This is in line with Cheng et al., who performed computational simulations on four patients with acute aortic dissection and suggested that larger entry tears and large percentage flows into the false lumen were associated with poor short-term outcomes [37]. Furthermore, as we decided to treat the artery wall as a rigid structure, and clinical data from specific patients were applied together with calculated Reynolds number below the turbulent level, we decided to characterize blood flow as a laminar, which was in line with Lo et al. Doutel et al., who investigated large arteries reconstructed for patient-specific data [38,39].

Finally, computational simulations in patient-specific models have shown highly turbulent flow in aortic dissections, leading to areas of abnormal wall shear stress.

### Limitation to the Study

The benefits of CFD must be viewed in the context of known limitations. First, the sample size was relatively small, and most analyses included a cohort of less than 30 patients. Moreover, simulations may not be that specific to the individual patient given the continuous physiological fluctuations, which are affected by a host of factors, such as lifestyle, medication, or genetic predisposition. Therefore, integration of patient-specific data is lacking and should be addressed in the future.

## 5. Conclusions

The proposed numerical approach allowed the reconstruction of blood flow before and after an aorta’s wall dissection. CFD model indicated that places with a lower value of blood velocity and shear rate correlated with higher blood viscosity and a probability of thrombus appearance. Moreover, with an increase in *Hct*, blood viscosity also increased, while the intensity of blood flow provoked changing viscosity values in these areas. Furthermore, the velocity gradient near the tear surface caused high wall WSS, which could lead to the decreased resistance in the aorta’s wall with further implication to a patient.

## Figures and Tables

**Table 1:** Spatial characterization of analyzed patients. Dissection and prosthesis placement according to Fillinger et al., 2010 [20]. P—proximal; LSA—left subclavian artery; SG—stent-graft; SGS—self-expanded stents; LRA—left renal artery; RIA—right iliac artery; Z—zone; R—renal; I—iliac.

Name	P1	P2	P3	P4	P5	P6	P7	P8	P9	P10	P11	P12
Dissection Type	III b	III b	III b	III b	III b	III b	III b	III b	III b	III b	III b	III b
Entry Tear	P	P	P	P	P	P	P	P	P	P	P	P
	LSA	LSA	LSA	LSA	LSA	LSA	LSA	LSA	LSA	LSA	LSA	LSA
End of Dissection	RIA (Z:9)	RIA (Z:9)	RIA (Z:9)	RIA (Z:9)	RIA (Z:9)	RIA (Z:9)	LRA (Z:8)	LRA (Z:8)	LRA (Z:8)	LRA (Z:8)	LRA (Z:8)	LRA (Z:8)
Vascular Prosthesis	SG	SG	SG	SG	SG	SG	SG	SG	SG	SG	SG	SG
	117 mm	117 mm	117 mm	117 mm	115 mm	115 mm	115 mm	115 mm	115 mm	115 mm	115 mm	115 mm
	(Z:3-4)	(Z:3-4)	(Z:3-4)	(Z:3-4)	(Z:3-4)	(Z:3-4)	(Z:3-4)	(Z:3-4)	(Z:3-4)	(Z:3-4)	(Z:3-4)	(Z:3-4)
	SGS (LRA-Z:10; R-25 mm; RIA-Z:8; I-25 mm)	SGS (LRA-Z:10; R-25 mm; RIA-Z:8; I-25 mm)	SGS (LRA-Z:10; R-25 mm; RIA-Z:8; I-25 mm)	SGS (LRA-Z:10; R-25 mm; RIA-Z:8; I-25 mm)	SGS (LRA-Z:10; R-25 mm)	SGS (LRA-Z:10; R-25 mm)	SGS (LRA-Z:10; R-25 mm)	SGS (LRA-Z:10; R-25 mm)	SGS (LRA-Z:10; R-25 mm)	SGS (RIA-Z:8; I-25 mm)	SGS (RIA-Z:8; I-25 mm)	SGS (RIA-Z:8; I-25 mm)

## Data Availability

The data used to support the findings of this study are available from the corresponding author upon request.
